# SIMAP—the database of all-against-all protein sequence similarities and annotations with new interfaces and increased coverage

**DOI:** 10.1093/nar/gkt970

**Published:** 2013-10-26

**Authors:** Roland Arnold, Florian Goldenberg, Hans-Werner Mewes, Thomas Rattei

**Affiliations:** ^1^Terrence Donnelly Centre for Cellular and Biomolecular Research, Kim Lab, University of Toronto, Toronto, ON M5S 3E1, Canada, ^2^CUBE—Division of Computational Systems Biology, Department of Microbiology and Ecosystem Science, University of Vienna, 1090 Vienna, Austria and ^3^Institute of Bioinformatics and Systems Biology, Helmholtz Zentrum München, Technische Universität München, Wissenschaftszentrum Weihenstephan, 85764 Neuherberg, Germany

## Abstract

The Similarity Matrix of Proteins (SIMAP, http://mips.gsf.de/simap/) database has been designed to massively accelerate computationally expensive protein sequence analysis tasks in bioinformatics. It provides pre-calculated sequence similarities interconnecting the entire known protein sequence universe, complemented by pre-calculated protein features and domains, similarity clusters and functional annotations. SIMAP covers all major public protein databases as well as many consistently re-annotated metagenomes from different repositories. As of September 2013, SIMAP contains >163 million proteins corresponding to ∼70 million non-redundant sequences. SIMAP uses the sensitive FASTA search heuristics, the Smith–Waterman alignment algorithm, the InterPro database of protein domain models and the BLAST2GO functional annotation algorithm. SIMAP assists biologists by facilitating the interactive exploration of the protein sequence universe. Web-Service and DAS interfaces allow connecting SIMAP with any other bioinformatic tool and resource. All-against-all protein sequence similarity matrices of project-specific protein collections are generated on request. Recent improvements allow SIMAP to cover the rapidly growing sequenced protein sequence universe. New Web-Service interfaces enhance the connectivity of SIMAP. Novel tools for interactive extraction of protein similarity networks have been added. Open access to SIMAP is provided through the web portal; the portal also contains instructions and links for software access and flat file downloads.

## INTRODUCTION

Protein sequences are ubiquitous study subjects in molecular biology. They are determined in large quantities by sequencing of genomic DNA followed by the computational prediction of coding regions or mapping of additional data from functional genomics. During the past decades, protein sequence databases accumulated many millions of different protein sequences, representing blueprints of the function and structure of the encoded gene products. However, many protein sequences are ‘hypothetical’ by nature as their sequences have never been experimentally confirmed and their cellular functions cannot be rationally predicted except by information transfer from known and evolutionary related proteins. Therefore, the comprehensive computational characterization of an increasing proportion of the protein sequence universe is a never-ebbing spring of experimentally testable research hypotheses and one of the central tasks of computational biology (1).

Basic approaches such as pairwise sequence similarity-based searches [e.g. BLAST (2)] or comparisons of protein sequences against secondary databases of protein families [e.g. InterPro (3)] still play an outstanding role within the huge repertoire of computational methods inferring evolutionary relationships between proteins and predicting functional attributes. They are frequently used by individuals for querying public sequence databases but also build the basis for the comprehensive prediction of protein clusters (4), orthologs and paralogs (5–8) or functional annotations (9–11). An increasing number of computational tools use protein similarity networks to illustrate functional relationships between huge groups of proteins (12–15). The rapidly increasing number of publicly available protein sequences escalates the computational costs related to these bioinformatics tasks, particularly if they require all-against-all calculations of sequence similarities or sequence features. For largest-scale projects [e.g. (7)], the calculation of a sequence matrix between all proteins easily outgrows available computational resources.

The Similarity Matrix of Proteins (SIMAP) database solves the computational dilemma described above by incrementally pre-calculating the sequence similarities interconnecting the entire known protein sequence universe (16). SIMAP implements an incremental update strategy that efficiently integrates newly published protein sequences. It uses idling CPU power of many thousand computers contributed by volunteers in the BOINCSIMAP public resource computing network (17). The initial concept of SIMAP was pre-calculating sequence similarities based on the FASTA (18) search heuristics and the Smith–Waterman alignment algorithm (19), restricted by a static and sensitive raw score threshold (≥80; BLOSUM50) without limiting the maximal number of hits per sequence. Later on we extended SIMAP and also included pre-calculated protein domains and features, functional annotations, clusters and pre-annotated metagenomes (20–22).

The similarity-network representation of the known protein universe by SIMAP turned out to be a versatile and powerful tool in sequence analysis. Here we describe three representative use cases of the SIMAP database:

- *Interactive exploration of the protein sequence universe:* Individual users identify proteins of interest by text- or sequence-based searches on the SIMAP web interface. For each protein, SIMAP immediately lists potential homologs based on sequence similarity or domain architecture similarity. Homologs can be restricted to selected taxa and sequence databases; results can be displayed according to their scores as well as in a taxonomic tree. Every match found can be used as starting point for subsequent SIMAP queries.
- *Acceleration of large-scale sequence similarity calculations, e.g. in genome annotation:* Genome-wide sequence similarity searches against different databases can be replaced by SIMAP database lookups, using the Web-Service or DAS programmatic interfaces. The search space of each query is specifically defined by selection of taxonomic lineages and protein databases. Sequence similarities, sequence alignments, protein domain annotations, cluster and function predictions can be retrieved. If a query sequence is not already known to SIMAP (occurs rarely due to its high coverage), either the rapid SIMAP SeqFinder (20) can be used to identify its most similar sequence for querying SIMAP or this sequence can be analysed independent from the SIMAP matrix. This strategy works successfully, enabling the PEDANT database to cover and annotate all RefSeq genomes (23).
- *Project-specific preparation of all-against-all protein sequence similarity matrices:* Owing to the tremendous volume of the entire SIMAP database, it is most practicable that all-against-all protein sequence similarity matrices of project-specific protein collections are extracted on request from SIMAP and provided for download. Such protocol has been used multiple times, e.g. in case of the STRING database (24) and consists of three phases (import of the project-specific protein collection into SIMAP; calculation of sequence similarities and domains for sequences that are new to SIMAP; extraction and transfer of the project-specific submatrix).

SIMAP is not the only database developed for pre-calculated sequence similarities and protein domains. Compared with alternative approaches such as NCBI BLink (25) and EBI CluSTr (26), SIMAP provides more flexible access to users as well as significantly higher coverage with respect to the number of proteins and the number of links stored. The pre-calculated protein domains provided by the InterPro consortium (3) cover the UniProt proteins and are thereby a subset of SIMAP, which covers many more proteins, e.g. from NCBI RefSeq (27) or from metagenome projects (22).

Recent improvements of the SIMAP storage infrastructure allowed us to keep up with the rapidly growing protein sequence universe, which results in even faster growth of the SIMAP database owing to the quadratic complexity of the all-against-all sequence similarity matrix. To facilitate the integration of SIMAP into other bioinformatic projects and workflows, we have improved the data access facilities and added novel tools to SIMAP for interactive extraction of protein networks based on sequence similarity or domain architecture similarity.

## NEW FEATURES AND IMPROVEMENTS IN SIMAP

### Covering the growing protein sequence universe

SIMAP monthly synchronizes its protein repository with all major public sequence databases. As demonstrated earlier (21), the contents of these databases considerably differ and no pair completely resembles each other. The diversity of annotations has even increased over the past years, particularly for genomes of model organisms and higher eukaryota. SIMAP has therefore also integrated databases specifically focusing on re-annotation of genomes, such as ENSEMBL (28) and ENSEMBL GENOMES (29). SIMAP typically imports and processes 0.5–1 million additional non-redundant sequences per month (as by 2013). Table 1 lists the contents of SIMAP by September 2013. The pre-calculated sequence similarity matrix in SIMAP grows proportional to the squared number of non-redundant protein sequences. Data are stored as sorted adjacency lists in binary compressed flat files indexed by the file system. Although each hit only occupies ∼10 bytes, the total matrix occupies ∼60 TB of disk space. To keep the access performance high, even with the rapidly increasing size of SIMAP, we have migrated these data to a high-performance array of six parallel storage units. Currently, SIMAP processes up to 2 million queries per day on the internal middleware level including on average 50 000 individual requests per day over the Web portal and other interfaces.

**Table 1.** Number of protein entries, non-redundant sequences, pre-calculated sequence similarities, protein domains, features and functional annotations (all given in millions) in SIMAP as of September 2013

The protein sequence universe covered by SIMAP	Protein entries:	163
	Unique sequences (non-metagenomic):	27
	Unique sequences (metagenomic):	35
Sequence similarities	FASTA/Smith-Waterman hits	3 517 306
InterPro hits	BlastProDom	1
	FPrintScan	28
	HMMPanther	40
	HMMPfam	50
	HMMPIR	2
	HMMSmart	16
	HMMTigr	7
	ProfileScan	17
	PatternScan	10
	Superfamily	39
	Gene3D	43
	Coil	8
	Seg	71
	HAMAP	2
Sequence features	SignalP	30
	TargetP	51
	TMHMM	39
	PHOBIUS	45
Functional annotations	Blast2GO	157

### Redesigned Web-Service interfaces

**Figure 1.**
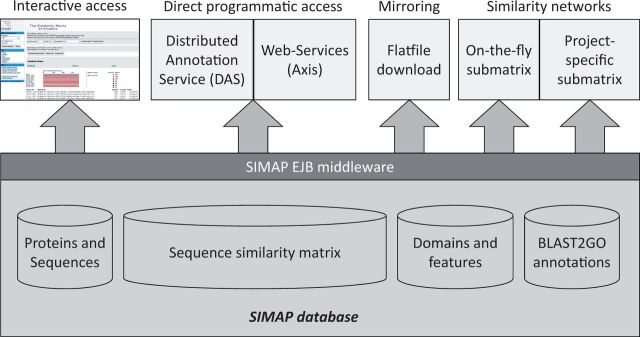
Schematic representation of the SIMAP database contents and access facilities.

To date, several bioinformatics resources use SIMAP as basis for further analysis as for the creation of orthologous groups (7). Other resources integrate SIMAP result lists directly in their online reports on proteins of interest as GeneCards (30) or CYGD (31). Currently, these systems mainly use bulk download of SIMAP data, which needs to be updated with each release. However, in those cases where individual SIMAP results for proteins are needed to create web-based dynamic information, up-to-date data can be fetched using the SIMAP Web-Service capabilities. We implemented the service using the latest version of Axis 2 to ensure optimal stability and performance. Currently, the Web-Service allows to fetch for a given sequence all instances in the primary databases, to lookup of pre-calculated Interpro hits and sequence features, and to retrieve of homology information in either the SIMAP internal XML schema or formatted as BLAST XML output. We extensively tested the Web-Service for stability and performance. Even for a geographically remote client, the response times for each query are below 2 s (tested from Toronto, Ontario, Canada). An overview on the performance measurements for methods with a sequence as input can be found in Table 2. The link to the Web-Service including detailed instructions and an example Java client can be found on the SIMAP main portal http://mips.gsf.de/simap/. Figure 1 summarizes and illustrates the overall structure of the SIMAP database contents and access facilities.

**Table 2.** Performance of the main methods of the SIMAP Web-Service

Web-Service method	Request per minute from a single client
Retrieval of homologs (SIMAP XML)	26
Retrieval of homologs (BLAST XML)	25
Retrieval of InterPro hits	37

Values denote average numbers of requests per minute from a geographically remote location (Toronto, Ontario, Canada).

A Web-Service client can be easily generated using code generation utilities, which are available for most programming languages as the perl SOAP::Lite module, and SIMAP queries can then seamlessly be integrated into work flows or analysis systems on the user side. The typical use case starts with a sequence of interest for which data should be fetched. Since SIMAP internally uses unique MD5 hashes to refer to a sequence object, all Web-Service queries that refer to a protein as input use a md5 hash as a key. The routines to compute this checksum are available in all major programming languages. To keep the amount of sequences or domain hits reasonable, the client has to specify the maximal E-value, raw-score cut-offs and a maximal upper bound of hits. Also, the search space can be set by the client for homology searches, which eases the selection of hits relevant for a given project. To define a search space, the system allows filtering for certain taxonomical branches (by giving lists of NCBI taxonomy database IDs to either include or exclude) or primary database IDs as used in SIMAP internally. These database IDs can be fetched by an own Web-Service method. The client retrieves an XML result string that can be either parsed for the information wanted or be processed using an XML transformation system as XSLT for, e.g., display on a web page.

### Submatrices: direct access to the protein similarity network

An increasing number of computational tools make use of sequence similarity networks between selected collections of protein sequences. These networks are shaped by evolutionary processes and are crucial for the inference of protein functions. For small numbers of proteins, similarity networks can be calculated on-the-fly (13). The computational complexity of all-against-all comparisons, however, restricts this approach. SIMAP addresses all use cases that need sequence similarity networks of medium (genome-scale) or large (database-scale) size. Whereas large similarity networks for many millions of proteins are specifically generated on request, small and medium networks can be extracted interactively from SIMAP as ‘submatrices’. These networks need a careful selection of representative node proteins, e.g. too many highly related proteins impair the visual representation of similarity networks. If proteins are clustered into redundant groups, representatives might be selected by their taxonomic affiliation or functional annotation. SIMAP therefore provides three principal modes for submatrix downloads:

- User-defined selection of protein sequences: The nodes of the network are selected according to a protein file, containing either names or sequences. This mode is beneficial for users who have specifically pre-clustered proteins and selected representatives.
- User-defined selection criteria: The nodes are selected automatically by SIMAP based on selection criteria such as taxonomic affiliation and originating database. This mode is able to generate genome-wide similarity networks [e.g. between all human proteins from the NCBI RefSeq database (27)].
- User-defined central protein: The user defines the central node by its protein name or sequence. Optionally, further nodes can be restricted by taxonomic and database selection criteria. SIMAP determines the direct and indirect neighbours (up to a user-defined number of links) of the central node and constructs the sequence similarity network between them.

The density of the resulting sequence similarity network can be individually controlled by different parameters (such as e-Value, bitScore, number of hits) in all modes. If, according to the requested parameters, the resulting network would be too large for direct access via the Web Portal, an information page is displayed suggesting contacting the SIMAP staff to request the project-specific generation of the subnetwork.

### Pre-calculated domain architecture similarities

The representation and specific arrangement of domains in protein sequences provide additional highly valuable information for the evolutionary and functional analysis of protein sequences and ideally complement pairwise sequence similarities. Despite their bias towards protein families well represented in public databases, domains are used for both fast and sensitive protein similarity searches [e.g. (32)]. SIMAP offers two tools supporting this strategy.

SIMAP pre-calculated Interpro (3) domains for the entire protein universe covered, including all metagenomes. Owing to a unique incremental update facility for the InterPro models, updating SIMAP to a new InterPro release is computationally efficient and can be performed with a total calculation time of ∼1 week. All pre-calculated domains are provided in flat files for download.

SIMAP provides domain architecture similarities for interactive exploration in its Web portal. All proteins in SIMAP are linked to their domain architecture similarity report, listing domain-based homologs according to the user-defined selection of InterPro member database and e-Value threshold. To facilitate large-scale projects, we now also provide full dumps of the non-redundant domain architectures for all InterPro member databases linked to all SIMAP proteins for download.

## OUTLOOK

The SIMAP database will continue to update its contents monthly. The computational costs for the resulting sequence similarity calculations are considerable, but can be well covered by the BOINCSIMAP project (17). Further consolidation of data storage facilities will soon be necessary owing to the rapidly growing size of the raw sequence similarity data (currently 60 TB). Migration of the SIMAP data to cloud-based storage is therefore planned, but will require project-specific adaptations to ensure high performance both for reading and writing (incorporating data from monthly updates). Data retrieval will be further improved by the replacement of the previous middleware that will allow even higher performance for all query types.

Furthermore, we have already planned to increase the sensitivity and accuracy of the SIMAP matrix using composition-based score adjustment (33,34) that is so far not used in SIMAP and will switch from FASTA (18) search heuristics fully to the non-heuristic Smith–Waterman algorithm (19). This will require a recalculation of the whole matrix; therefore, it will be performed in multiple steps (whole-genome and full-length proteins first, metagenomes thereafter).

## FUNDING

Funding for database development and operation, University of Vienna. Funding for open access charge: University of Vienna.

*Conflict of interest statement.* None declared.
